# A blood-based immune marker for resistance to pembrolizumab in patients with metastatic urothelial cancer

**DOI:** 10.1007/s00262-022-03250-0

**Published:** 2022-08-17

**Authors:** Maud Rijnders, Debbie G. J. Robbrecht, Astrid A. M. Oostvogels, Mandy van Brakel, Joost L. Boormans, Maureen J. B. Aarts, Hayri E. Balcioglu, Paul Hamberg, Jens Voortman, Hans M. Westgeest, Martijn P. Lolkema, Ronald de Wit, Astrid A. M. van der Veldt, Reno Debets

**Affiliations:** 1 Departments of Medical Oncology, Erasmus MC Cancer Institute, Erasmus University Medical Center, Dr. Molewaterplein 40, 3015 GD Rotterdam, The Netherlands; 2 Department of Urology, Erasmus MC Cancer Institute, Erasmus University Medical Center, Rotterdam, The Netherlands; 3 Department of Medical Oncology, GROW School for Oncology and Developmental Biology, Maastricht University Medical Center+, Maastricht, The Netherlands; 4 Department of Medical Oncology, Franciscus Gasthuis & Vlietland Hospital Rotterdam and Schiedam, Rotterdam, The Netherlands; 5 Department of Medical Oncology, Amsterdam UMC, Vrije Universiteit Amsterdam, Cancer Center Amsterdam, Amsterdam, The Netherlands; 6 Department of Medical Oncology, Amphia Hospital Breda, Breda, The Netherlands; 7 Radiology & Nuclear Medicine, Erasmus MC Cancer Institute, Erasmus University Medical Center, Rotterdam, The Netherlands

**Keywords:** Metastatic urothelial cancer, Cancer immunotherapy, PD1 inhibition, Translational medical research, Biomarkers, Flow cytometry

## Abstract

PD1 inhibition is effective in patients with metastatic urothelial cancer (mUC), yet a large fraction of patients does not respond. In this study, we aimed to identify a blood-based immune marker associated with non-response to facilitate patient selection for anti-PD1. To this end, we quantified 18 immune cell populations using multiplex flow cytometry in blood samples from 71 patients with mUC (as part of a biomarker discovery trial; NCT03263039, registration date 28-08-2017). Patients were classified as responder (ongoing complete or partial response, or stable disease; *n* = 25) or non-responder (progressive disease; *n* = 46) according to RECIST v1.1 at 6 months of treatment with pembrolizumab. We observed no differences in numbers of lymphocytes, T-cells, granulocytes, monocytes or their subsets between responders and non-responders at baseline. In contrast, analysis of ratios of immune cell populations revealed that a high mature neutrophil-to-T-cell ratio (MNTR) exclusively identified non-responders. In addition, the survival of patients with high versus low MNTR was poor: median overall survival (OS) 2.2 vs 8.9 months (hazard ratio (HR) 6.6; *p* < 0.00001), and median progression-free survival (PFS) 1.5 vs 5.2 months (HR 5.6; *p* < 0.0001). The associations with therapy response, OS, and PFS for the MNTR were stronger than for the classical neutrophil-to-lymphocyte ratio (HR for OS 3.5, and PFS 3) and the PD-L1 combined positivity score (HR for OS 1.9, and PFS 2.1). In conclusion, the MNTR distinctly and uniquely identified non-responders to treatment and may represent a novel pre-treatment blood-based immune metric to select patients with mUC for treatment with pembrolizumab.

**Supplementary Information:**

The online version contains supplementary material available at 10.1007/s00262-022-03250-0.

## Background

The therapeutic landscape of metastatic urothelial cancer (mUC) has changed since immune checkpoint inhibitors (ICI) directed against programmed cell death protein (PD)1 or its ligand (PD-L1) were introduced. ICIs are approved in the first- (1, 2) and second-line setting (3, 4) for mUC, as maintenance therapy for patients who had a response to chemotherapy (5), and for treatment of *Bacillus Calmette-Guérin*-unresponsive *carcinoma *in situ of the bladder (6). Overall, response rates of ICIs in mUC are modest, and given the high costs and accompanying toxicities (7) pre-treatment selection of patients is critical.

Currently, PD-L1 expression in tumor tissue is the only approved biomarker that is used for selection of cisplatin-ineligible patients for ICIs in the first-line setting (8, 9). In the second-line setting, PD-L1 expression does not have predictive value (3), and no biomarkers are applied yet for patient selection. In an effort to identify new selection markers, previous studies have revealed that numbers of circulating T-cells at baseline, and dynamic changes in particular T-cell subsets during treatment, are associated with response to ICIs (10–13). Furthermore, a relationship between the neutrophil-to-lymphocyte ratio (NLR) and clinical outcome of patients has been observed in multiple tumor types, including UC (14–17). However, these studies generally have shortcomings, such as lack of predictive value, use of immune cell fractions rather than numbers, and focus on on-treatment rather than baseline predictors. In the current study, we have enumerated 18 immune cell populations in blood of 71 patients with mUC by multiparameter flow cytometry, to study whether individual immune cell populations or ratios thereof identify patients with mUC who do or do not respond to pembrolizumab.

## Methods

### Patients and assessment of clinical response

Patients with locally advanced or mUC of the bladder or upper urinary tract with an indication for pembrolizumab were included in a phase II prospective biomarker discovery study (NCT03263039), and treated as described previously (pembrolizumab, 200 mg intravenously, 3-weekly (18)). Patients were classified as responder (ongoing complete or partial response, or stable disease) or non-responder (progressive disease) at 6 months after treatment initiation according to response evaluation criteria in solid tumors (RECIST) v1.1. Overall survival (OS) was defined as the time from start of pembrolizumab to date of death; progression-free survival (PFS) was defined as the time from start of pembrolizumab to clinical or radiological disease progression.

### Multiplex flow cytometry of blood samples

Peripheral blood was prospectively collected in EDTA tubes at baseline, and weeks 6 and 12 of treatment. Whole blood was stained and analyzed by multiplex flow cytometry to quantify 18 immune cell populations as described previously (19), antibody specifications are listed in Supplementary Table 1. In short, lymphocyte, T-cell, granulocyte, and monocytes populations were gated separately in a scatter plot of CD45+ staining versus side scatter. Immune cell populations were further defined using the following markers for B cells: CD3- CD19+ ; natural killer (NK) cells: CD3- CD56+ CD16± ; T-cells: CD3+ ; γδ T-cells: CD3+ TCRγδ+ ; CD4 or CD8 T-cells: CD3+ TCRγδ- CD4+ or CD8+ ; eosinophils: CD15+ CD16-; mature neutrophils: CD15^high^ CD16^high^; immature neutrophils: CD15+ CD16+ ; classical monocytes: CD14+ CD16-; intermediate monocytes: CD14+ CD16+ ; non-classical monocytes: CD14- CD16+ ; dendritic cells (DC): CD14- CD16- CD11c+ ; and myeloid derived suppressor cells (MDSC): CD14+ CD16- CD11b+ HLA-DR^low^. Besides quantitation of immune cell populations for individual timepoints, we performed normalization of data to more specifically assess longitudinal changes in numbers of immune cell populations. To this end, the measured numbers were normalized per patient by subtracting the patients’ mean number for a given population from the individual measurement, followed by addition of the overall mean number of that particular population.

### PD-L1 immunohistochemistry and scoring

The PD-L1 combined positivity score (CPS) was determined on fresh metastatic tumor biopsies obtained prior to start of therapy (n = 46), or archival tumor tissue (n = 25), using the companion diagnostic assay of pembrolizumab (PD-L1 IHC 22C3 pharmDx, Agilent Technologies, Carpinteria, CA, USA).

### Multiplex immunofluorescence of tumor tissue

Multiplex immunofluorescence was performed using OPAL reagents (Akoya Biosciences, Marlborough, MA, USA) on 4-μm sections of FFPE tumor biopsies as described previously (18). The sequence of antibody stainings was as follows: 1. CD4 (FP1600/EP204, Akoya Biosciences, 1:100) – OPAL520; 2. CD8 (FP1601/144B, Akoya Biosciences, 1:200) – OPAL690; 3. CD66b (80H3, Sanbio, 1:100) – OPAL570; 4. Cytokeratin-Pan (AE1/AE3, Invitrogen, 1:500) – Opal 620; and 5. DAPI. Digital image analysis was also performed as described previously (18). Cellular densities were calculated by dividing the number of cells with a certain phenotype by the total area of that region and were averaged per patient across all regions of interest. Distances from a cell with a certain phenotype to the nearest cell with another phenotype were calculated by nearest neighbor analysis; this was averaged across all cells, and per patient across all regions of interest.

### Statistical analysis

Statistical analysis was performed using R version 3.5.1. Use of the Mann–Whitney U, Wilcoxon signed rank, or Fisher’s exact test is specified in figure legends. The optimal cut-off level for dichotomous analysis of immune markers was determined using receiver operating characteristic (ROC) curves. OS and PFS were estimated using Kaplan–Meier estimates, patients who were alive or without disease progression were censored at last date the patient was known to be alive, or at last date of tumor assessment. Hazard ratios (HR) were calculated using univariate Cox regression models. Multivariate Cox regression analysis was performed for known risk factors: performance status, hemoglobin concentration, presence of liver metastases, and time since completion of previous treatment. Correction for multiple testing was performed using the Holm-Bonferroni method.

## Results

### Patient cohort

In this study, 71 patients with mUC received first- (*n* = 9) or second-line (*n* = 62) treatment with pembrolizumab. Non-responders were younger than responders and had a lower albumin concentration in blood (Table 1). For patients who received first-line pembrolizumab a PD-L1 CPS of  ≥ 10 was required; five of these patients were responders. In the second-line setting, 55% of responders versus 29% of non-responders had a positive PD-L1 CPS (Table 1).

**Table 1:** Patient characteristics of 71 patients with metastatic urothelial cancer

	Responders *n* = 25	Non-responders *n* = 46	*p*-value
Age—median (range) ‡	72 (41–85)	67 (29–78)	**0.03**
Male gender—no. (%)§	21 (84%)	30 (65%)	0.09
Primary tumor location—no. (%)§			
Bladder	18 (72%)	26 (57%)	0.31
Upper urinary tract	4 (16%)	17 (37%)	0.10
Both	3 (12%)	3 (7%)	0.66
Prognostic factors—no. (%)§			
ECOG performance score of 1^1^	16 (64%)	29 (63%)	1.0
Lymph node-only disease	9 (36%)	9 (20%)	0.15
Liver metastases	4 (16%)	16 (35%)	0.10
Lactate dehydrogenase concentration > 248 U/L	6 (24%)	13 (28%)	0.79
Hemoglobin concentration < 10 g/dL	19 (76%)	40 (87%)	0.32
Albumin concentration < 35 mg/L	0	7 (15%)	**0.047**
Treatment-free interval < 3 months from previous chemotherapy	4 (20%)	14 (33%)	0.38
Treatment line—no. (%)§			
First-line	5 (20%)	4 (9%)	0.262
Second-line	20 (80%)	42 (91%)	
PD-L1 combined positivity score^2^—no. (%)§			
Positive (≥ 10) in first-line patients	5 (100%)	4 (100%)	1.0
Positive (≥ 10) in second-line patients	11 (55%)	12 (29%)	0.054

Patients were stratified according to response to pembrolizumab at 6 months of therapy (responders: ongoing complete or partial response, or stable disease; non-responders: progressive disease). ^1^Eastern cooperative oncology group performance status, score of 0 or 1 was required. ^2^PD-L1 expression in tumor tissue according to the companion diagnostic assay of pembrolizumab. ‡Mann–Whitney U test, §Fisher’s Exact test

No differential numbers of immune cell populations in blood of non-responders versus responders to pembrolizumab at baseline.

**Fig. 1:**
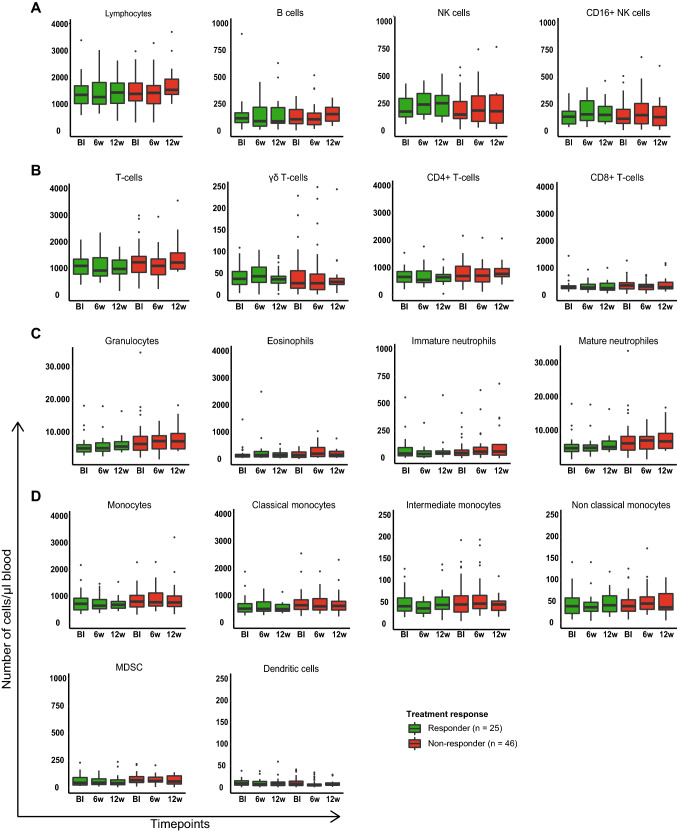
Numbers of immune cell populations in blood were not different between responders and non-responders to pembrolizumab at baseline and during treatment. Boxplots display the number of cells belonging to subsets of: **a** lymphocytes; **b** T-cells; **c** granulocytes; and **d**. monocytes per microliter blood. Immune markers per subset are provided in the methods section. Timepoints: baseline (Bl), 6w, 12w (6, 12 weeks of treatment). Differences between responders and non-responders were determined using the Mann–Whitney U test, and differences between timepoints were determined for paired samples using the Wilcoxon signed rank test, *p*-values were corrected for multiple testing using the Holm-Bonferroni method

Fresh blood samples were available for 71 patients at baseline (*n* = 26 responders, *n* = 45 non-responders), for 55 patients at week 6 (*n* = 21 responders, *n* = 34 non-responders), and for 38 patients at week 12 (*n* = 22 responders, *n* = 16 non-responders). At baseline, no differences were observed between responders and non-responders in the numbers (number of cells per µl blood) of lymphocytes and their subsets (B cells, NK cells, and CD16+ NK cells; Fig. 1a), T-cells and their subsets (T-cells, γδ T-cells, CD4+ T-cells, and CD8+ T-cells; Fig. 1b), granulocytes and their subsets (eosinophils, immature neutrophils, and mature neutrophils; Fig. 1c), nor monocytes and their subsets (classical monocytes, intermediate monocytes, non-classical monocytes, MDSC, and DC; Fig. 1d). Also at weeks 6 and 12 of therapy, the numbers of all 18 immune cell populations in blood remained non-different between responders and non-responders (Fig. 1a–d). To specifically assess therapy-induced changes, numbers of immune cell populations were normalized per patient (see methods section). We did not observe on-treatment changes in any of the immune cell populations either in responders or non-responders (Supplementary Fig. 1).

The mature neutrophil-to-T-cell ratio at baseline exclusively identifies non-responders to pembrolizumab.

**Fig. 2:**
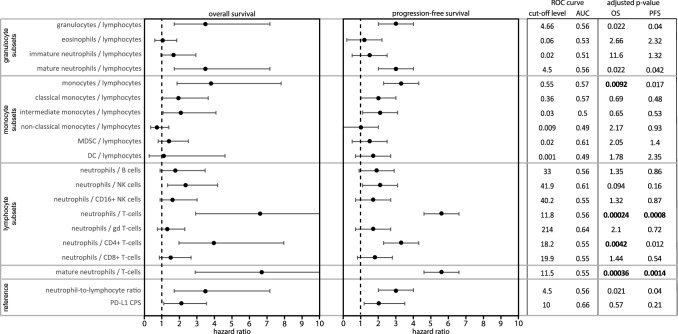
Testing ratios of granulocyte, monocyte and lymphocyte subsets demonstrated that the mature neutrophil-to-T-cell ratio was most strongly associated with overall and progression-free survival. Forest plots displaying hazard ratios (HR) for overall (OS; left) and progression-free survival (PFS; right) for ratios of immune cell populations. The classical neutrophil-to-lymphocyte ratio and PD-L1 combined positivity score (CPS; bottom two rows) were used as references. Plot displays from left to right: the optimal cut-off level for survival analyses based on receiver operating characteristics (ROC); the area under the curve (AUC); and adjusted p-values for OS and PFS. P-values were corrected for multiple testing using the Holm-Bonferroni method. No significant associations with OS and PFS were observed when a median split was used for survival analyses

**Fig. 3:**
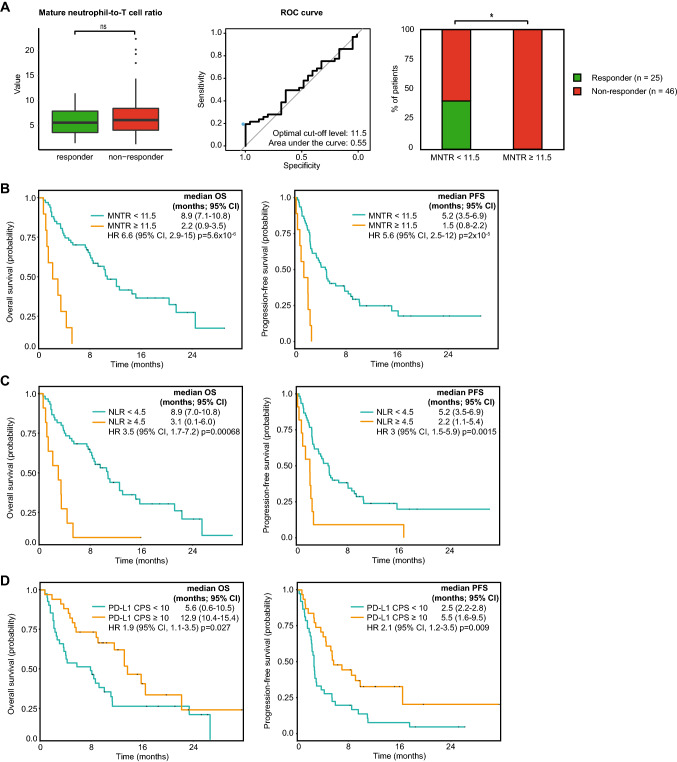
High mature neutrophil-to-T-cell ratio at baseline distinctly identified non-responders to pembrolizumab. **a** Left graph: boxplot displaying the mature neutrophil-to-T-cell ratio (MNTR) in responders and non-responders at baseline. Middle graph: receiver operating characteristic (ROC) curve for MNTR. Right graph: fraction of responders and non-responders in patients with MNTR < 11.5 (*n* = 62) and ≥ 11.5 (*n* = 9). **b** Kaplan–Meier estimation of overall survival (OS; left graph) and progression-free survival (PFS; right graph) for patients with MNTR < 11.5 and ≥ 11.5. **c** Kaplan–Meier estimation of OS (left graph) and PFS (right graph) for patients with NLR < 4.5 and ≥ 4.5. **d** Kaplan–Meier estimation of OS (left graph) and PFS (right graph) for patients with PD-L1 combined positivity score (CPS) < 10 (n = 39) and ≥ 10 (n = 32). CI: confidence interval. HR: hazard ratio

Since numbers of individual immune cell populations were not distinctive between responders and non-responders at baseline, we systematically interrogated ratios of granulocyte, monocyte and lymphocyte subsets, and assessed their association with OS and PFS at their respective optimal cut-off levels (Fig. 2). We assessed the classical NLR, defined as the quotient of the sum of mature and immature neutrophil counts and the sum of total lymphocyte and T-cell counts, and the PD-L1 CPS, as references. For granulocyte subsets, the ratio of mature neutrophils to lymphocytes showed similar associations with OS and PFS as the classical NLR, whereas no associations were observed for immature neutrophils or eosinophils. For monocyte subsets, the ratio of monocytes to lymphocytes was associated with OS and PFS. For lymphocyte subsets, the strongest association with OS and PFS was observed for the ratio of mature neutrophils to T-cells (MNTR), which was mostly attributed to CD4+ and not CD8+ T-cells. The median value of the MNTR was not different between responders and non-responders (Fig. 3a, left graph). When using an optimal cut-off level of 11.5 (Fig. 3a, middle graph), this ratio exclusively identified non-responders (*n* = 9; Fig. 3a, right graph). The positive predictive value (PPV) of a high MNTR for non-response to therapy was 100%, with a specificity of 100% and sensitivity of 19%. Thereby the MNTR outperforms the classical NLR (PPV 91%, specificity 96%, sensitivity 22%) and the PD-L1 CPS in the total cohort (PPV 50%, specificity 36%, sensitivity 35%). Patients with a high versus low MNTR had a significantly shorter OS (median 2.2 vs 8.9 months; HR 6.6; *p* = 5.6 × 10^–6^) and PFS (median 1.5 vs 5.2 months; HR 5.6; *p* = 2 × 10^–5^; Fig. 3b). This association with survival was stronger compared to the classical NLR (Fig. 3c) and PD-L1 CPS (Fig. 3d). Finally, multivariate cox regression analysis revealed that MNTR was the strongest factor associated with OS (*p* < 0.0001) and PFS (*p* < 0.0001), and a weaker association was observed for presence of liver metastases (*p* = 0.02 for OS, and not significant for PFS), and a treatment-free interval of less than three months from previous chemotherapy (*p* = 0.021 for OS, and *p* = 0.03 for PFS).

## Discussion

In this study, we enumerated 18 immune cell populations in prospectively collected fresh blood samples from 71 patients with mUC treated with pembrolizumab and demonstrated that a high MNTR prior to treatment is associated with therapy resistance. These data introduce a new blood-based immune marker that can be measured easily and non-invasively, and that has the potential to identify patients with mUC who will not benefit from pembrolizumab before treatment initiation.

The presented data extend our previous study on the frequency of T-cell subsets in blood samples of 56 out of these 71 patients with mUC (18). In this earlier study we demonstrated that responders harbor higher frequencies of CD4+ T-cells that express PD1 and 4-1BB when compared to non-responders at baseline, and responders showed changes in frequencies of these subsets during treatment. In the current study, we analyzed numbers of 14 additional immune cell populations in blood. We did not identify differences between responders and non-responders for any of the 18 T-cell, lymphocyte, granulocyte or monocyte populations at baseline nor at weeks 6 and 12 of treatment. Moreover, we did not identify longitudinal changes during therapy for any of these immune cell populations in responders or non-responders. In line with previous studies (14–17), we did show that the classical NLR is related to OS and PFS. Extending our analyses to novel ratios of immune cell populations, revealed that the quotient of mature neutrophils and T-cells outperformed the classical NLR and PD-L1 CPS, and was superior above  all ratios regarding its potency to discriminate non-responders from responders, and regarding its association with Os and PFS.

From a mechanistic point of view, the negative predictive value of the MNTR may be in line with earlier reports showing that tumor-infiltrating neutrophils form a barrier around tumor cells, and as such prevent adequate contact between tumor cells and T-cells (20). When studying paired tumor biopsies from patients with a high versus low MNTR, however, we did not observe differences in densities of CD4+ T-cells, CD8+ T-cells or CD66b+ neutrophils, nor differences in distances among these cells (Supplementary Fig. 2A-D). In other words, we cannot support a direct relationship between a high MNTR in blood with tumor cell-entrapment by neutrophils in tumor tissue. Previously, we showed that a lack of CD4+ T helper type 1 (Th1) cells in the tumor at baseline, and their inability to cluster with CD8+ T-cells and myeloid cells upon treatment, were associated with resistance to pembrolizumab (18). Also, in case of the MNTR, it appeared that lack of CD4+ rather than CD8+ T-cells was predominantly associated with non-response and limited survival. Exclusion of CD8+ T-cells from the MNTR, however, reduced its association with survival, suggesting that involvement of CD8+ T-cells in this metric is not negligible. Future studies in patient blood and tumor specimens are required to identify the underlying mechanism of action.

The MNTR can be measured non-invasively by a commonly used technique (flowcytometry) that comes with a low cost burden. Identification of patients with a high MNTR may prevent patients with mUC from receiving potentially toxic and ineffective treatment with pembrolizumab. The optimal cut-off level for MNTR was determined specifically for this study and may therefore overestimate survival associations. Along this line, maximally selected rank statistics (20) were employed as an alternative approach to determine the optimal cut-off levels, and yielded similar results (data not shown). Our results require validation in an independent cohort of patients with mUC treated with an ICI; however, to the best of our knowledge, a homogeneous cohort of patients with thorough measurements of numbers of immune cell populations in blood, is currently not available.

In conclusion, we showed that a high MNTR at baseline is associated with treatment resistance and poor OS and PFS. This new blood-based marker potentially enables stratification of patients with mUC for treatment with pembrolizumab, thereby improving clinical outcomes and quality of life while reducing costs of care.

### Supplementary Information

Below is the link to the electronic supplementary material.

Supplementary Fig. 1. Responders and non-responders to pembrolizumab do not demonstrate longitudinal changes in normalized numbers of immune cell populations in blood. Boxplots display the normalized number of cells belonging to subsets of: **a** lymphocytes; **b** T-cells; **c** granulocytes; and **d** monocytes per microliter blood (see Methods section for details on normalization and staining methods). Timepoints: baseline (Bl), 6w, 12w (6, 12 weeks of treatment). Differences between timepoints were determined for paired samples using the Wilcoxon signed rank test and p-values were corrected for multiple testing using the Holm-Bonferroni method. (PDF 618 KB)

Supplementary Fig. 2. Patients with low versus high mature neutrophil-to-T-cell ratio in blood do not show differences in tissue contexture of neutrophils and T-cells at baseline. **a** Representative multiplex immunofluorescence image of a lymph node metastasis. Tissue sections were stained for CD4+ T-cells (green), CD8+ T-cells (red), CD66b+ neutrophils (orange), and pan-cytokeratin (CK) positive tumor cells (cyan; see Methods section for details). **b** Densities (cells/mm2) as well as **c** ratios of densities and **d** distances (in µm) among CD4+ T-cells, CD8+ T-cells, CD66b+ neutrophils, and CK+ tumor cells were displayed for patients with a low (< 11.5) versus high mature neutrophil-to-T-cell ratio (MNTR ≥ 11.5). None of the differences were statistically significant (Mann–Whitney U test; *p*-values were corrected for multiple testing using the Holm-Bonferroni method). (PDF 3706 KB)

Supplementary Table 1. Overview of antibodies used for flow cytometry. (PDF 98 KB)

## Data Availability

Anonymized patient data, and flow cytometry data are available upon request. Requests can be send to Prof. dr. Reno Debets, Laboratory of Tumor Immunology, Department of Medical Oncology, Erasmus MC-Cancer Institute, Rotterdam, The Netherlands.
