# Supplementary figures and images for: A blood-based immune marker for resistance to pembrolizumab in patients with metastatic urothelial cancer
Source: Cancer Immunol Immunother. 2022 Aug 17;72(3):759–67. doi: 10.1007/s00262-022-03250-0 (PMC9947015; doi:10.1007/s00262-022-03250-0)

Supplementary figure 1

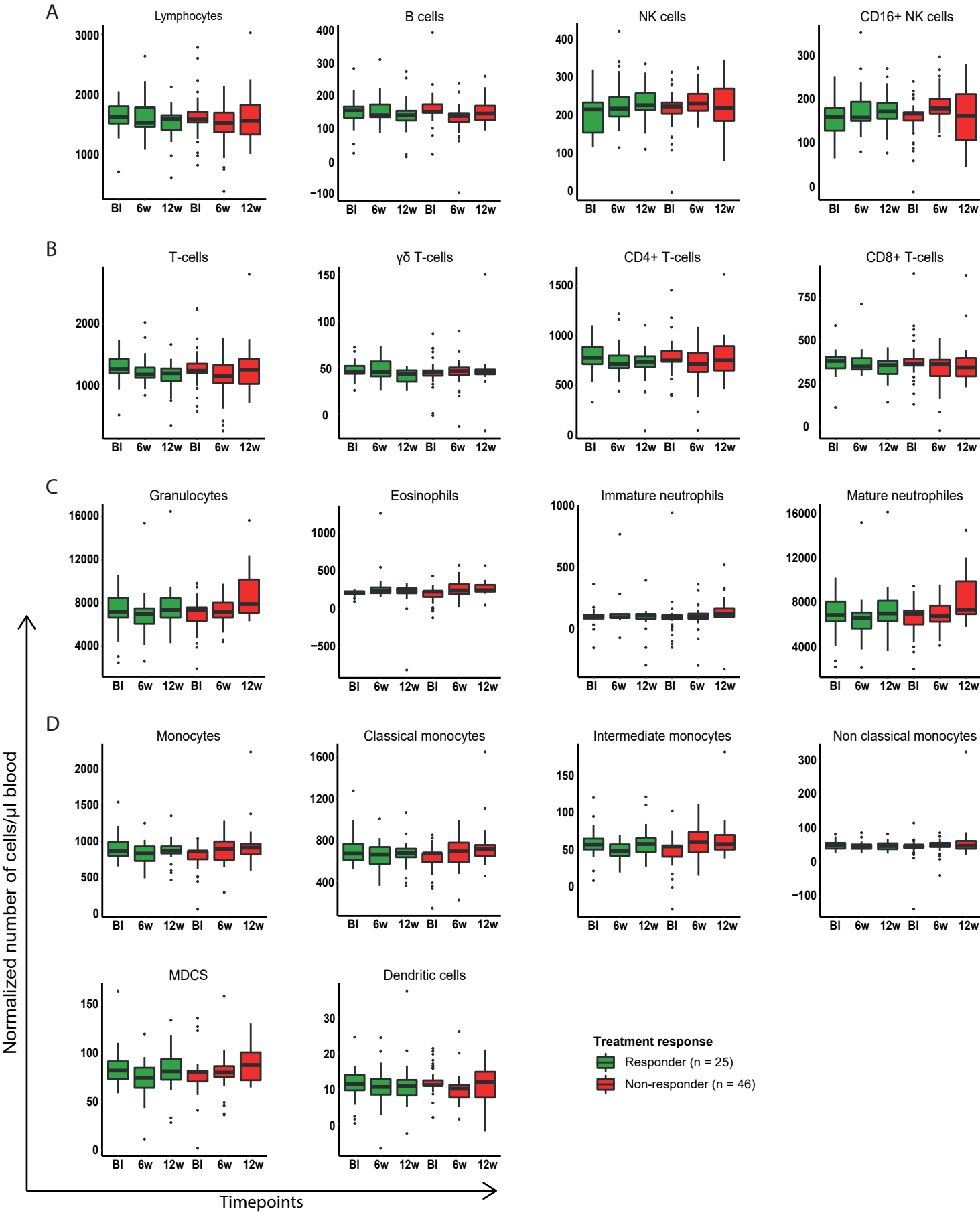

Supplement: Supplementary file 1 — Supplementary Fig. 1. Responders and non-responders to pembrolizumab do not demonstrate longitudinal changes in normalized numbers of immune cell populations in blood. Boxplots display the normalized number of cells belonging to subsets of: a lymphocytes; b T-cells; c granulocytes; and d monocytes per microliter blood (see Methods section for details on normalization and staining methods). Timepoints: baseline (Bl), 6w, 12w (6, 12 weeks of treatment). Differences between timepoints were determined for paired samples using the Wilcoxon signed rank test and p-values were corrected for multiple testing using the Holm-Bonferroni method. (PDF 618 KB) [file 262_2022_3250_MOESM1_ESM.pdf]

Supplementary figure 2

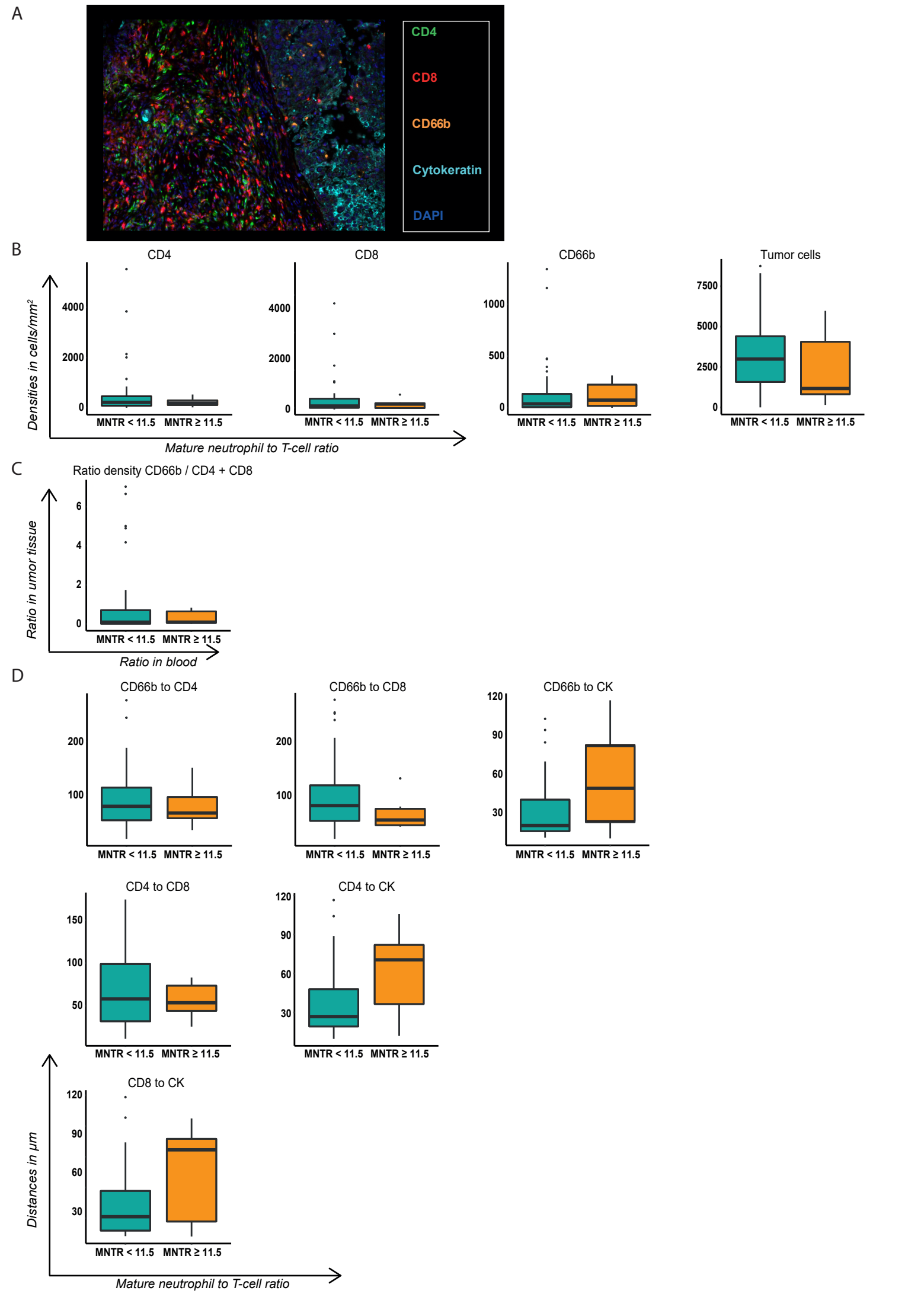

Supplement: Supplementary file 2 — Supplementary Fig. 2. Patients with low versus high mature neutrophil-to-T-cell ratio in blood do not show differences in tissue contexture of neutrophils and T-cells at baseline. a Representative multiplex immunofluorescence image of a lymph node metastasis. Tissue sections were stained for CD4+ T-cells (green), CD8+ T-cells (red), CD66b+ neutrophils (orange), and pan-cytokeratin (CK) positive tumor cells (cyan; see Methods section for details). b Densities (cells/mm2) as well as c ratios of densities and d distances (in µm) among CD4+ T-cells, CD8+ T-cells, CD66b+ neutrophils, and CK+ tumor cells were displayed for patients with a low (< 11.5) versus high mature neutrophil-to-T-cell ratio (MNTR ≥ 11.5). None of the differences were statistically significant (Mann–Whitney U test; p-values were corrected for multiple testing using the Holm-Bonferroni method). (PDF 3706 KB) [file 262_2022_3250_MOESM2_ESM.pdf]
