# Supplementary material for: A blood-based immune marker for resistance to pembrolizumab in patients with metastatic urothelial cancer
Source: Cancer Immunol Immunother. 2022 Aug 17;72(3):759–67. doi: 10.1007/s00262-022-03250-0 (PMC9947015; doi:10.1007/s00262-022-03250-0)
Supplement: Supplementary file 3 — Supplementary Table 1. Overview of antibodies used for flow cytometry. (PDF 98 KB) [file 262_2022_3250_MOESM3_ESM.pdf]

| Antibody | Clone | Supplier | Dilution |
|---|---|---|---|
| CD3-PB | UCHT1 | BD Biosciences | 1:50 |
| CD4-V500 | RPA-T4 | BD Biosciences | 1:50 |
| CD8-APC-Cy7 | SK1 | BD Biosciences | 1:200 |
| CD11b-APC | D12 | BD Biosciences | 1:40 |
| CD11c-APC-eF780 | BU15 | eBioscience | 1:50 |
| CD14-FITC | M ϕP9 | BD Biosciences | 1:20 |
| CD15-PE | HI98 | BD Biosciences | 1:20 |
| CD16-PE-Cy7 | 3G8 | BD Biosciences | 1:400 |
| CD19-V500 | HIB19 | BD Biosciences | 1:50 |
| CD45-PerCP | 2D1 | BD Biosciences | 1:50 |
| CD56-APC | TULY56 | eBioscience | 1:40 |
| HLA-DR-BV786 | G46-6 | BD Biosciences | 1:80 |
| TCRgd-FITC | 11F2 | BD Biosciences | 1:20 |

**Supplementary Table 1. Overview of antibodies used for flow cytometry.**
